# Protocol for a feasibility registry-based randomised controlled trial investigating a tailored follow-up service for stroke (A-LISTS)

**DOI:** 10.1186/s40814-024-01527-y

**Published:** 2024-07-30

**Authors:** Dominique A. Cadilhac, Andrew G. Ross, Kathleen L. Bagot, Jannette M. Blennerhassett, Monique F. Kilkenny, Joosup Kim, Tara Purvis, Karen M. Barclay, Fiona Ellery, Julie Morrison, Jennifer Cranefield, Timothy J. Kleinig, Rohan Grimley, Katherine Jaques, Dana Wong, Lisa Murphy, Grant Russell, Mark R. Nelson, Vincent Thijs, Colin Scott, Sandy Middleton, the A-LISTS investigator group

**Affiliations:** 1 Stroke Theme, The Florey Institute of Neuroscience and Mental Health, Heidelberg, VIC Australia; 2 Stroke and Ageing Research, Department of Medicine, School of Clinical Sciences at Monash Health, Monash University, Melbourne, VIC Australia; 3 College of Sports, Health and Engineering, Victoria University, Melbourne, Australia; 4 Physiotherapy Department and Health Independence Program, Austin Health, Heidelberg, VIC Australia; 5 Department of Nursing & Allied Health, School of Health Sciences, Swinburne University of Technology, Hawthorn, Vic Australia; 6 Care Economy Research Institute, La Trobe University, Bundoora, Australia; 7 Department of Neurology, Royal Adelaide Hospital, Adelaide, SA Australia; 8 School of Medicine and Dentistry, Griffith University, Birtinya, QLD Australia; 9 Medicine, Sunshine Coast University Hospital, Birtinya, Australia; 10 Wesley Mission Queensland, Brisbane, QLD Australia; 11 School of Psychology and Public Health, La Trobe University, Bundoora, Melbourne, VIC Australia; 12 Stroke Foundation, Melbourne, VIC Australia; 13 Department of General Practice, Faculty of Medicine, Nursing and Health Sciences, School of Public Health and Preventative Medicine, Monash University, Melbourne, VIC Australia; 14 Menzies Institute for Medical Research, University of Tasmania, Hobart, TAS Australia; 15 Department of Neurology, Austin Health, Melbourne, VIC Australia; 16 Melbourne Medical School, University of Melbourne, Melbourne, VIC Australia; 17 Stroke Association of Victoria, Melbourne, VIC Australia; 18 Nursing Research Institute, St Vincent’s Health Network Sydney and Australian Catholic University, Sydney, NSW Australia

**Keywords:** Stroke, Clinical trial protocol, Follow-up service, Clinical Quality Registry

## Abstract

**Background:**

Stroke affects long-term physical and cognitive function; many survivors report unmet health needs, such as pain or depression. A hospital-led follow-up service designed to address ongoing health problems may avoid unplanned readmissions and improve quality of life.

**Methods:**

This paper outlines the protocol for a registry-based, randomised controlled trial with allocation concealment of participants and outcome assessors. Based on an intention-to-treat analysis, we will evaluate the feasibility, acceptability, potential effectiveness and cost implications of a new tailored, codesigned, hospital-led follow-up service for people within 6–12 months of stroke. Participants (*n* = 100) from the Australian Stroke Clinical Registry who report extreme health problems on the EuroQol EQ-5D-3L survey between 90 and 180 days after stroke will be randomly assigned (1:1) to intervention (follow-up service) or control (usual care) groups. All participants will be independently assessed at baseline and 12–14-week post-randomisation. Primary outcomes for feasibility are the proportion of participants completing the trial and for intervention participants the proportion that received follow-up services. Acceptability is satisfaction of clinicians and participants involved in the intervention. Secondary outcomes include effectiveness: change in extreme health problems (EQ-5D-3L), unmet needs (Longer-term Unmet Needs questionnaire), unplanned presentations and hospital readmission, functional independence (modified Rankin Scale) and cost implications estimated from self-reported health service utilisation and productivity (e.g. workforce participation). To inform future research or implementation, the design contains a process evaluation including clinical protocol fidelity and an economic evaluation.

**Discussion:**

The results of this study will provide improved knowledge of service design and implementation barriers and facilitators and associated costs and resource implications to inform a future fully powered effectiveness trial of the intervention.

**Trial registration:**

ACTRN12622001015730pr.

**Trial sponsor:**

Florey Institute of Neuroscience and Mental Health, 245 Burgundy Street, Heidelberg, VIC, 3084, PH: +61 3 9035 7032

**Supplementary Information:**

The online version contains supplementary material available at 10.1186/s40814-024-01527-y.

## Background

Stroke is a leading cause of global disease burden [1]. In addition to the immediate physical, cognitive and emotional injury impacts post-stroke, the long-term effects can be significant and life-altering. Approximately, 25% of people with stroke report their quality of life as equivalent to, or worse than, death [2]. Physical disability, loss of employment, social isolation, cognitive impairment, communication difficulties, anxiety and depression make resuming home and community activities difficult [3]. Furthermore, compared with hospital discharges to rehabilitation or aged care, people discharged directly home are at an increased risk of an unplanned readmission within 90 days (sub-hazard ratio, 1.44 [95% *CI*, 1.33–1.55]) [4]. Further, one in five people living with stroke have no support services in place after discharge from hospital [4].

Data from the Australian Stroke Clinical Registry (AuSCR) describe significant impacts on people with stroke or transient ischaemic attack (TIA) between 90 and 180 days after hospital admission [5, 6]. For example, health-related quality-of-life data (EQ-5D-3L, EuroQoL 5-dimensions 3-level version) [7] demonstrated that patients reported some or extreme problems with mobility (50%), self-care (30%), usual activities (58%), pain/discomfort (49%) and anxiety or depression (49%) [7]. To help mitigate these reported problems, efforts to better integrate care across hospital and primary care settings for chronic diseases such as stroke are required.

Evaluation from follow-up services in other countries is promising [8–10]. For example, a stroke nurse navigator programme in the United States reduced 30-day unplanned readmissions by 67.6% [10]. In addition, several authors of different studies have reported positive findings for follow-up services delivered from 30 to 90 days within the Australian context [11, 12]. In the study by Pugh et al., when compared with usual care, the nurse-led model of transitional care for neurology patients discharged from hospital produced cost savings, a positive return on investment, improved functional status and health-related quality of life [12]. In a separate feasibility study for the use of a modified World Stroke Organization post-stroke checklist in a rehabilitation setting, the authors reported improved communication with patients and timely referrals to appropriate clinical services [11]. Although these studies suggested that 60% of people living with stroke still had a least one health problem at 3-month follow-up [12], no studies have focused on providing follow-up support after 3 months.

More efficient and targeted approaches that include better communication between hospital specialist services and primary care providers are required for people living with stroke in Australia [13]. Furthermore, some stroke impacts may only become apparent post-discharge, and community-based services may lack the expertise to address stroke-related problems. To address this important gap in stroke care, we codesigned a registry-based, hospital-led tailored follow-up service with key stakeholders and people with lived experience as part of the AuSCR *LI*fe after *S*troke *T*ailored *S*upport (A-LISTS) study [14]. The follow-up service includes an intervention package that comprises a clinical protocol and procedure manual to be used by the site service coordinators (hereafter service coordinator/s) to tailor the support provided to the individuals with identified unmet need(s) [14]. The service coordinator is a nominated stroke clinician — nurse or allied health staff — who is trained in the procedures. The newly developed follow-up service intervention package was pilot tested in one urban hospital in Australia with six participants and then refined based on feedback from the service coordinator and participants to ensure it was ready to be used in a feasibility randomised controlled trial (RCT).

## Methods

### Research aims

The aim of the study is to assess the feasibility (i.e. acceptability and satisfaction of service coordinators and participants), potential clinical effectiveness, participant resource utilisation and cost implications of the tailored hospital-led follow-up service for chronic stroke compared with usual care (control).

### Study design

**Fig. 1:**
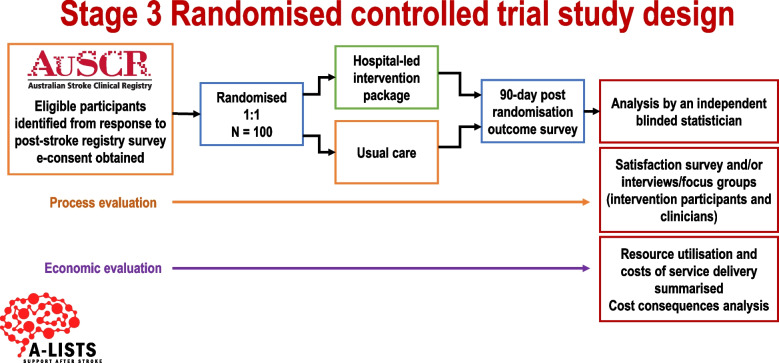
Overview of study design

Multicentre, registry-based, trial with a prospective, parallel, randomised controlled, two group, single-blinded design (Fig. 1) with an intention-to-treat analysis. The RCT has been prospectively registered with Australian New Zealand Clinical Trials Registry (ACTRN12622001015730p, 20th July 2022).

Methods and results will be reported in compliance with the CONSORT 2010 statement, including the extension for randomised pilot and feasibility trials [15]. A process evaluation and an economic evaluation will be conducted concurrently to the main trial and will be reported using the relevant reporting guidelines (e.g. CHEERS checklist) [16]. The study database will be created via REDCap (Research Electronic Data Capture; a secure web based data management system) [17] and will be hosted on the Florey Institute of Neuroscience and Mental Health servers under the security and information technology infrastructure of the University of Melbourne, and will be protected as per industry standards.

### Study setting

Up to six eligible and interested hospitals from a variety of settings will be identified from the network of hospitals that participate in the AuSCR (*n* = 63 hospitals). Commencement dates will be staggered due to the timing of receiving hospital governance approvals, onboarding and training procedures. The AuSCR is a national clinical quality registry that collects prospective data on all patients from participating hospitals with a clinical diagnosis of stroke or TIA with the purpose of monitoring and improving stroke care in Australia [18]. The diagnosis is confirmed by the registrant when they complete a follow-up survey at 90–180 days. Within the AuSCR, data on demographics, clinical characteristics and evidence-based therapies provided to patients during the acute admission are collected by hospital staff. Registrants are then contacted by the AuSCR Office, initially by mail to complete a follow-up health outcome survey. Where there is no response, a short message service (SMS) and/or mail to the nominated next of kin is sent between 90 and 180 days post admission. The AuSCR Office attempts to obtain health outcomes from all registrants unless they request no follow-up, opt out of having their personal details stored on the registry, were registered on the AuSCR over 180 days post discharge, or were known to be deceased. At the 90–180 day follow-up, registrants provide information about their living situation and provide health-related quality of life (HRQoL) details using the EQ-5D-3L survey including the visual analogue scale (VAS) [19]. Functional independence is collected using the modified Rankin Scale (mRS) [20], and participants are also asked to indicate their willingness to be contacted for further research opportunities.

### Study population

#### Inclusion criteria

Participants are selected from the AuSCR registrants if they:

- Have indicated a willingness to be contacted for future research at 90–180 day follow-up
- Are aged ≥ 18 years with a confirmed diagnosis of stroke
- Are living in the community in a private residence
- Have reported an extreme problem in at least one dimension of the EQ-5D-3L or have a score on the VAS ≤ 60 at 90–180 day follow-up [19]
- Are able to participate in English and provide informed consent (self-report or appropriate proxy can assist).

#### Exclusion criteria

AuSCR registrants with a TIA diagnosis will be excluded. Registrants in palliative care and/or a residential aged care facility will be excluded as they may be unlikely to survive to the end of study follow-up period (i.e. 12–14-week post-randomisation).

### Trial procedures

#### Registry-based participant recruitment procedure

**Fig. 2:**
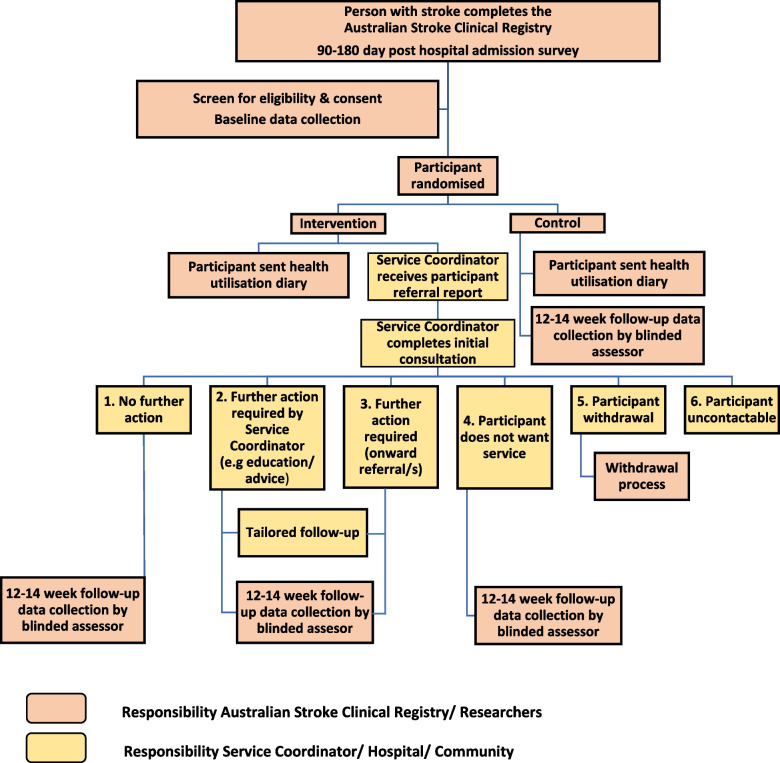
Summary of the recruitment and intervention pathway

Identification of eligible AuSCR registrants and recruitment will be undertaken by the data manager located at AuSCR Office in Melbourne, Victoria. Trained AuSCR team members will call potential participants to confirm eligibility. Consent will be obtained via mail (paper form) or email (e-consent; purpose designed in the REDCap database). Baseline assessments (including demographic and clinical data) will be completed by an AuSCR team member via telephone post consent. Figure 2 outlines the recruitment pathway. A screening log will be used to capture demographic information on consenting eligible registrants and those who are not, to enable reporting of response and participation rates.

#### Randomisation, allocation concealment and blinding procedure

Once consented, the trial participants will be randomly allocated 1:1 to the intervention group or control (usual care) group. Online randomisation will occur using REDCap [17], stratified by age (< 65, 65 + years) and sex (male, female) to ensure balance of age and sex between the two groups as these factors are associated with differences in HRQoL [21]. Participants and outcome assessors (AuSCR staff) will be blinded to the group allocation. Service coordinators delivering the intervention will be unaware of participants from their hospital randomised to the control group. To avoid unblinding to group allocation, the control group will not know which group they are in. As part of the study, some participants may be contacted by a member of staff from the hospital or service that treated them for their stroke.

#### Outcome measures

The primary and secondary outcomes are listed in Table 1 for each study aim. Data collection tools and relevant timepoints of measurement for the clinical, process and economic evaluation are outlined in Table 2.

**Table 1:** Primary and secondary aims and trial outcome measures

**Primary aims**	**Primary Outcomes**
Trial feasibility	
Acceptability	The proportion of participants that: • Complete the feasibility trial • Attend the follow-up service (intervention group)
Participant, service coordinator and clinician satisfaction^a^	Satisfaction and experiences of participants and service coordinators (+ / − other clinicians at participating health services) with the hospital-led follow-up service, assessed through project documentation, field notes, surveys and/or interviews/focus groups
**Secondary aims**	**Secondary outcomes**
Potential clinical effectiveness, i.e. health status	Health-related quality of life (EQ-5D-3L survey [19]) including change in the proportion with extreme health problems at 12–14 week post-randomisation. Also composite outcome: extreme health problems reported on EQ-5D-3L [19] or visual analogue scale score 60 points or less
	Unmet needs: Longer-Term Unmet Needs Survey [22]
	Unplanned emergency department presentations and/or admissions to hospital (self-reported)^b^
	Disability (modified Rankin scale [20])
Healthcare service utilisation^b^	Use of health and community services (self-reported)
	Change in medications (self-reported)
Cost implications of the programme^b^	Costs of intervention delivery (self-reported) Cost or cost offsets from health and community services used (self-reported)

^a^Will be included in the process evaluation

^b^Will be included in the economic evaluation and converted to costs

**Table 2:** Data collection tools for clinical, process and economic evaluation across trial timepoints

**Project stage**	**Measure**	**Measurement tool**	**Completed by**	**Pre-trial, eligibility**	**Baseline**	**During 12-week intervention**	**12–14 weeks-outcome assessment**	**Post-trial**
Identifying eligible participants	Ongoing health problems impacting quality of life, disability/dependence and living situation^a^	AuSCR follow-up survey including EQ-5D-3L [19], modified Rankin Scale (mRs) [20] and current residence	AuSCR registrant 90–180 days poststroke	X				
Clinical evaluation	Quality of life^b^	EQ-5D-3L (baseline: to verify responses from pre-trial is still current, and eligibility is still met for the service)	All participants in conjunction with blinded AuSCR follow-up team member		X		X	
	Disability/dependence^b^	mRS (baseline: to verify responses from pre-trial is still current, and eligibility is still met for the service) Communication and fatigue (yes/no and 5-point Likert scale, respectively)			X		X	
	Unmet needs	Longer-Term Unmet Needs for Stroke questionnaire [22]			X		X	
	Unplanned hospital readmissions, ED visits^b^	Survey tailored for A-LISTS trial			X		X	
	Serious adverse events^b^	Serious adverse events form specific for A-LISTS trial				X	X	
	Health service use diary for participants	Developed for trial to support recall; used as memory aid at the outcome assessment to respond to questions about current health services used	All participants			X		
Process evaluation	Satisfaction & experiences with service and study participation	Theory-informed survey and/or semi-structured interview/focus groups	Intervention participants				X	
	Satisfaction with service, including implementation features & intervention fidelity	Theory-informed survey and semi-structured focus groups [23, 24]	Service coordinator and clinicians					X
	Participation numbers, recommended actions, uptake of service coordinator recommendations	Template tailored for A-LISTS study; patient-level data will be aggregated	Service coordinator			X		
	Project documentation	Standard operating procedures, minutes, training documents, project notes	Project team, investigators	X	X	X	X	X
Economic evaluation	Current health services used, medications, costs^b^ Programme delivery costs	Resource use questionnaire developed for trial Research (financial) documents, invoices			X		X	

*Abbreviations*: *AuSCR* Australian Stroke Clinical Registry, *EQ-5D-3L* EuroQol 5-dimension 3-level survey; *mRS*, modified Rankin Scale

^a^Standard Australian Stroke Clinical Registry follow-up survey completed 90-180 days after admission

^b^indicates data will also be used for the economic evaluation

### Description of secondary outcome clinical effectiveness measures

Secondary outcome measures relating to the measurement of HRQoL, unmet needs and disability are described below.

#### HRQoL measured by EQ-5D-3L and Visual Analogue Scale

The EQ-5D-3L [19] is a standardised instrument developed by the EuroQol group to measure health-related quality of life and is widely used internationally and by the AuSCR. It comprises five dimensions: mobility, self-care, usual activities, pain/discomfort and anxiety/depression. Each dimension is self-reported by participants to indicate no problems, some problems or extreme problems. The EQ-5D-3L also includes a VAS. The VAS ranges from 0 (worst imaginable health state) to 100 (best imaginable health state). The median VAS reported by patients post-stroke in Australia is 70 [7], and a normative median score from a similar population without stroke is 80 [25].

#### Longer-term Unmet Needs after Stroke (LUNS) questionnaire

The LUNS questionnaire is a 22-item tool that enables the collection of the longer-term problems affecting the physical, psychological and social facets of people living with stroke [22]. It can also be used as a tool to evaluate community service usage and whether those community services are meeting the person’s needs [25]. Most studies have used the LUNS between 3 and 6 months post-stroke; however, it has been used up to 5–8 years following stroke [26]. The LUNS is acceptable to people living with stroke and has satisfactory validity and test–retest reliability [22].

#### Modified rankin scale (mRS) scale

The mRS is a single-item, global disability rating scale [20] often used in stroke trials for assessment of patient outcomes [27]. The categorical scale is as follows: 0 = no symptoms at all, 1 = no significant disability despite stroke-related symptoms, 2 = stroke-related disability but remains functionally independent, 3 = functionally dependent but independently mobile, 4 = requires assistance to mobilise and 5 = requires constant care and is bed-bound [28]. For this study, the mRS outcome will be dichotomised into two groups (0 to 2 [independent] vs dependent/dead [mRS 3 to 6]).

#### Sample size

Up to 100 people with stroke will be recruited (50 for intervention and 50 for control), which is consistent with recommendations for pilot and feasibility studies [29, 30]. It is anticipated that each participating hospital will provide the intervention to approximately 10–15 people, with a capacity of providing the follow-up service to 1–2 intervention participants per week.

#### Ethics

Ethics approval for this project was obtained by the Austin Health Human Research Ethics Committee (HREC/89487/Austin-2022). Hospital-specific governance approval will also be obtained from participating hospitals. Approval for the use of the existing AuSCR data has been obtained from the AuSCR Steering Committee, the governing body of AuSCR.

### Treatment groups

#### Intervention

Participants in the intervention group will receive the tailored follow-up service implemented over 12 weeks. The service coordinator will be provided with a tailored patient referral report (including demographic and clinical data collected from the baseline assessment). The intervention follow-up service utilises clinicians’ clinical reasoning and experience to help participants navigate the hospital, community and primary care systems. This support may include linking participants to appropriate locally available services. All service coordinators will have a clinical background in stroke (e.g. stroke nurse, stroke allied health) and will receive 4–8 hours of training tailored to the hospital setup. The service coordinator will conduct an initial assessment with the participant (either in-person or via telehealth; participant’s choice) to ascertain existing service usage and how to assist the participant with their unmet needs. Through a collaborative and shared decision-making approach with the participant, the level of input required will be tailored to the participant’s needs. The service coordinator will then organise referrals as required and provide advice and education as necessary.

Following the initial consultation, there are six possible scenarios (Fig. 2). Only existing and available services or treatments will be offered in the trial. The intervention follow-up service will be tailored to the individual participant. It does not dictate how often or which clinicians or services will have ongoing engagement with participants over the 12-week intervention period. Participants will be asked for their permission to share information with their general practitioner (GP) including the purposively designed A-LISTS GP letter. Depending on the participant’s age, location, needs, priorities and healthcare network, we envisage that some participants may be referred to services such as allied health services (e.g. physiotherapy, occupational therapy), community rehabilitation programmes and state-funded community health programmes (e.g. chronic disease management plan [31]). The service coordinator may also liaise with the National Disability Insurance Scheme [32], My Aged Care [33] and other stroke resources (e.g. Stroke Foundation services and information) to help participants navigate the system and provide education.

There will be no charges to participants for accessing the follow-up service, although some private services participants are referred to may incur fees. As this is an embedded real-world health services trial, we will utilise existing private, public, free and online services. Research funding will not be used to cover other out-of-pocket expenses (i.e. allied health services, specialist visits). Participants will be provided with an electronic or paper diary, to record health and community care contacts and referrals including dates and reasons for health and community care visits, to assist with completing the trial outcome assessment conducted at 12–14 weeks. The service coordinator is to record their notes as soon as possible after the service is delivered and ensure the letter using the template is sent to the GP.

#### Control group

Participants in the control group will receive their usual care (e.g. existing services or supports) in the community. They will also be provided with an electronic/paper diary to record health and community care contacts and referrals used to complete the outcome assessment conducted 12–14-weeks post-randomisation. At the end of the trial, information about participants in the control group who will be assessed as having ongoing high levels of unmet needs will be passed to the hospital team, who may choose to offer follow-up within current services available to them.

#### Safety monitoring

Occurrence of serious adverse events (SAEs) will be documented throughout the feasibility RCT by the service coordinator and blinded outcome assessor. Relevant information will be obtained from the participant and/or proxy and hospital medical records (where possible by the service coordinator accessing the medical records). SAEs are defined as any untoward or serious medical occurrence that results in death, life-threatening incidents, hospitalisations, an event that results in new disability/incapacity, or other important medical events [34]. SAEs will be reported to a neurologist who will act as the medical monitor (author V.T.) for adjudication. If the SAE is deemed to be related to the study intervention, then a report will be submitted to the ethics committee and the local research governance office.

#### Process evaluation

The process evaluation draws on implementation evaluation theory and models including the Medical Research Council guidance for complex interventions [35] and Normalisation Process Theory [36]. The process evaluation includes mixed methods since qualitative data in feasibility studies helps to refine the understanding of how the intervention works and facilitate ongoing adaptation of the intervention and evaluation design in preparation for a larger trial [37]. Data will be collected using project documentation, field notes, surveys and interviews/focus groups as outlined below.

#### Satisfaction survey

All participants will be invited to complete an electronic/paper satisfaction survey (including open and closed questions) at the 12–14-week post-randomisation outcome assessment. Information on satisfaction and experience with the care received in the community will be obtained, with specific questions related to the service coordinator and follow-up service also included for those in the intervention group. The service coordinator (and any other clinicians involved in the initial consultation) will also be invited to complete a survey exploring their experience of implementing and delivering the follow-up service.

#### Interviews/focus groups

At the conclusion of the RCT, semi-structured focus groups/interviews (*n* = 3, 6–10 in each group, with up to 30 people in total) will be undertaken with groups of the following: (i) clinicians involved in delivery of the service (all service coordinators and up to two other purposively selected clinicians per hospital if they were involved) and (ii) purposively selected participants, based on satisfaction variation from survey results, to further explore the facilitators and barriers to service implementation and delivery. Examples of questions are provided in Table 3.

**Table 3:** Example questions and prompts for focus-group interviews^a^

**Service co-ordinators and/or registry staff**	
**Question**	**Prompts**
Can you describe how the service was implemented at your site?	Were any aspects of the protocol provided changed for your site or for a specific clinician, health service or person with stroke?
Can you describe your experience with clinicians/people with stroke/health services/etc.?	Consider was it easy, difficult? Any policies or clinical pathways needing to be changed? Approvals required?
What resources were required to implement? Feedback on resources provided (request copies of changes to documents), including clarity of role and training	Consider information from project team, training, funds, time allocated vs required and other people to be involved?
Were any challenges experienced?	Did any clinician or person with stroke not want to participate? What concerns did they have? If they had concerns, how was this addressed? Were there any shared characteristics for participants withdrawing or refusing participation? Consider how could these be addressed? What resources/factors would be needed?
**Patient participants**	
**Question**	**Prompts**
Can you recall what you thought when you were first contacted by service?	What were your initial thoughts? What sorts of things were discussed?
Can you describe the experience of participating in this service overall?	What sorts of things occurred? Consider referrals, treatments and information
Did you have any concerns or difficulties with the service?	How were these addressed? What resources/factors would be needed?
Were there any benefits to you from participating in the service?	Consider all aspects of health and wellbeing What contributed? If any, do you think they will be maintained?
Anything important to improve the follow-up service?	Consider personnel, integration with existing care, information provided, referrals, accessibility of services and costs associated seeking recommended services or therapies

^a^This is not the full list of questions and prompts

Interviews/focus groups will be conducted remotely (e.g. telephone, video conference), recorded with participant consent and transcribed for analysis.

#### Economic evaluation

A cost consequences analysis will be undertaken to present disaggregated costs and outcomes of implementing the follow-up service [38]. This will clarify which costs and outcomes are most relevant to further refine the design of the service and a future effectiveness trial. Costs of providing the intervention will be estimated based on interviews with clinical leads at participating hospitals and from finance departments, where possible. The impacts of the intervention on resources used by participants will be estimated from a health sector (e.g. hospital presentations, general practitioner visits, specialist visits, outpatient visits) and societal perspective (e.g. employment, household productivity, informal care). Unit prices for resources used and productivity will be obtained from the most contemporary Australian sources. Data from participants will be self-reported, with a diary provided for the duration of the study to assist with collection of data related to health care resources utilised, for example use of health and community services, admissions to hospital or changes to medication (see also Table 1). This information will be supplemented by data from the follow-up service records of all referrals and service contacts for intervention participants. Medical records may also be audited to verify the data collected.

#### Statistical and data analyses

An independent statistician will conduct the analysis blinded to group allocation. Intention-to-treat and per protocol analyses (*participants who did not 'drop out' of service/withdraw or failed to attend service coordinator appointments*) will be described. Descriptive statistics will be reported for the participants’ characteristics, retention and completion of outcome measures by group allocation (intervention or control). The difference between groups for the primary outcome (completion of the feasibility trial) will be described as a difference in proportions. Other feasibility outcomes, including the proportion of intervention participants that attended the follow-up service, will be reported descriptively.

We acknowledge the limitations of between- and within-group comparisons of effectiveness in feasibility trials and the imprecision that small samples can create [39]. We will also assess within group changes to assess for minimum clinically important effects.

The EQ-5D-3L domains at pre-trial/baseline determined entry into the trial as people experiencing extreme health problems. We will describe the change in the proportion of participants with extreme health problems at 12 weeks between groups using the original criteria for entry into the trial. The EQ-5D-3L dimension responses will also be converted into a utility score using previously published algorithm for Australia [21]. Due to the anticipated skewed distribution of continuous health outcomes measures (e.g., utility values), between-group differences will be reported as median difference. Imputation of missing data will be undertaken as necessary. Multivariable median, logistic, and ordinal logistic regression models adjusted for baseline values to assess differences in health outcomes (e.g. VAS, EQ-5D-3L, LUNS, mRS) between groups. Confidence intervals will be reported for secondary health outcomes to inform discussion of the likely treatment effects of the intervention [40].

Open interview/focus-group transcripts, open-ended responses from the satisfaction surveys, and project documentation/field notes will be analysed using thematic and/or content analysis techniques. Both inductive and deductive methods may be incorporated as appropriate, within a framework analysis approach [41]. Ongoing discussions with the research team will be used to ensure the data are being interpreted and summarised to best reflect the intended meaning. Closed questions will be summarised descriptively. Use of triangulation, involving the combination of multiple data sources, methodological approaches and analysis methods [42], will be used to ensure comprehensiveness and encourage a more reflective analysis of the trial.

#### Prespecified criteria to judge proceeding with future definitive trial

Various aspects of this trial (participant-level data, process evaluation and economic evaluation data) will provide evidence to support the investigators in making changes to the protocol and in the determination of whether we proceed to a definitive trial based on the current or a modified format. For progression, we anticipate the following: recruit at least 25% of potentially eligible participants, consent at least 40% of people identified as eligible and who agreed to participate, > 80% retained, > 70% adherence to core study protocol components (i.e. intervention group participation in the initial consultation), complete data for 80% of primary and secondary health outcome surveys, at least 30% with a positive change in health status from baseline (i.e. fewer unmet needs or extreme problems reported at 12-week EQ-5D-3L), no serious adverse events related to the study intervention or other procedures, > 60% satisfied and would recommend the trial to others (with a larger proportion in the intervention group). Ability to recruit hospitals (> 70% that indicated willingness and progressed to site-specific ethics participated) and the resources to conduct a future trial will also be important criteria for progression.

## Discussion

The multicentre, hospital-led follow-up service (A-LISTS), is to be evaluated in this feasibility trial. The aim of the intervention is to support people experiencing stroke who report extreme health problems that have been identified using routinely collected national registry data within 3–6 months of a new stroke. The proposed intervention package was codesigned [14] and should support greater engagement of hospital clinicians, primary care and community-based services. This trial will enable insights into the various contextual factors that exist in the adoption of this type of registry-based, hospital-led service for stroke. Findings will provide improved knowledge of service design and implementation barriers and facilitators and associated costs and resource implications. The clinical health outcome data will support the calculation of potential effect sizes to inform planning a future fully-powered effectiveness trial of the intervention.

## Trial status

The trial has started with 50 participants randomised (1 May 2024) but had not finished recruiting when this version was submitted to the journal.

### Supplementary Information

Additional file 1. SPIRIT Checklist

## Data Availability

N/A.

## Abbreviations

A-LISTS: AuSCR LIfe after *S*troke Tailored Support
AuSCR: Australian Stroke Clinical Registry
EQ-5D-3L: EuroQoL 5-dimensions 3-level version
VAS: Visual Analogue Scale
GP: General Practitioner
HRQoL: Health-related quality of life
mRS: modified Rankin Scale
REDCap: Research Electronic Data Capture
SAE: Serious Adverse Events
TIA: Transient Ischaemic Attack
LUNS: Longer-term Unmet Needs after Stroke
